# The EMPRES-i genetic module: a novel tool linking epidemiological outbreak information and genetic characteristics of influenza viruses

**DOI:** 10.1093/database/bau008

**Published:** 2014-03-06

**Authors:** Filip Claes, Dmitry Kuznetsov, Robin Liechti, Sophie Von Dobschuetz, Bao Dinh Truong, Anne Gleizes, Daniele Conversa, Alessandro Colonna, Ettore Demaio, Sabina Ramazzotto, Fairouz Larfaoui, Julio Pinto, Philippe Le Mercier, Ioannis Xenarios, Gwenaelle Dauphin

**Affiliations:** ^1^Animal Health Service, Food and Agriculture Organization of the United Nations (FAO), Viale delle Terme di Caracalla, 10532 Rome, Italy and ^2^Vital-IT/Swiss-Prot Groups, SIB, Swiss Institute for Bioinformatics, Quartier Sorge, 1015 Lausanne, Switzerland

## Abstract

Combining epidemiological information, genetic characterization and geomapping in the analysis of influenza can contribute to a better understanding and description of influenza epidemiology and ecology, including possible virus reassortment events. Furthermore, integration of information such as agroecological farming system characteristics can provide new knowledge on risk factors of influenza emergence and spread. Integrating viral characteristics into an animal disease information system is therefore expected to provide a unique tool to trace-and-track particular virus strains; generate clade distributions and spatiotemporal clusters; screen for distribution of viruses with specific molecular markers; identify potential risk factors; and analyze or map viral characteristics related to vaccines used for control and/or prevention. For this purpose, a genetic module was developed within EMPRES-i (FAO’s global animal disease information system) linking epidemiological information from influenza events with virus characteristics and enabling combined analysis. An algorithm was developed to act as the interface between EMPRES-i disease event data and publicly available influenza virus sequences in OpenfluDB. This algorithm automatically computes potential links between outbreak event and sequences, which are subsequently manually validated by experts. Subsequently, other virus characteristics such as antiviral resistance can then be associated to outbreak data. To visualize such characteristics on a geographic map, shape files with virus characteristics to overlay on other EMPRES-i map layers (e.g. animal densities) can be generated. The genetic module allows export of associated epidemiological and sequence data for further analysis. FAO has made this tool available for scientists and policy makers. Contributions are expected from users to improve and validate the number of linked influenza events and isolate information as well as the quality of information. Possibilities to interconnect with other influenza sequence databases or to expand the genetic module to other viral diseases (e.g. foot and mouth disease) are being explored.

**Database OpenfluDB URL:** http://openflu.vital-it.ch

**Database EMPRES-i URL:** http://EMPRES-i.fao.org/

## Introduction

Both spatial and molecular data are important to the field of infectious disease epidemiology and ecology; these types of data should be considered within a common framework. Molecular tools provide new opportunities for understanding the ecology of infectious diseases, for example, by increasing our understanding of factors that determine spatial and temporal distribution of pathogens and disease (1, 2). Influenza virus segmented genome is highly dynamic and evolves through genetic shift or drift events (3). Phylogenetic analyses can elucidate the genetic origins, selection pressures, evolutionary rates, reassortment histories, population dynamics and migration patterns of influenza viruses in different host populations (4, 5). Given the complexity of influenza occurrence and circulation in time, location and hosts, these studies should rely on solid and associated molecular and epidemiological data.

To understand how, when and where animal influenza viruses may evolve into pandemic agents, monitoring the occurrence, evolution and spread of these viruses is needed as well as a better understanding of the factors that drive these aspects. The chain of events that builds up to a pandemic starts long before we are able to measure the first indications of an emergent pandemic virus. To be able to early detect progenitors with high potential risk to public health, there is therefore an absolute need to monitor a combination of both epidemiological and genetic aspects of influenza events in humans, swine and birds (6–9).

Nevertheless linkage between disease events and sequence information may be lost or remain uncertain for various reasons: the time lag between generation of the epidemiological information at field level and the genetic sequence in the laboratory, management of the information in different locations (databases) and by different technical teams (laboratory-epidemiology-public health-animal health), political barriers for the sharing of sensitive information related to disease outbreaks with potential trade implications, lack of awareness regarding the importance of keeping these two sets of information closely associated and finally, frequently insufficient allocated human resources for proper data recording. High-quality linkage requires close interactions between epidemiologists, laboratory specialists and bioinformaticians, and the development of computational methods capable of working with incomplete and uncertain data. To date, the only database that combines influenza epidemiological surveillance data with sequences is the Influenza Research Database (IRD, 10). FAO's official influenza epidemiological outbreak information is not linked to viral sequences by any web resource yet. In addition, molecular epidemiology is still at its infancy and there is no straightforward method for combining influenza epidemiological and virological data, even for modeling (11, 12) and risk assessment.

The genetic module presented in this article has been developed on the basis of interconnecting two databases, OpenFluDB (13) containing molecular data and EMPRES-i (14) containing epidemiological data.

The OpenFlu database (OpenFluDB) (15) is a publicly available influenza-specialized database that contains genomic and protein influenza virus sequences and related information. The isolate annotation includes virus type, host, geographical location, as well as other imported and generated data, e.g. putative enhanced pathogenicity and human adaptation, or antiviral resistance computed from protein sequences. The content of OpenFluDB is kept up-to-date by daily runs of an automatic procedure importing data from public repositories such as GenBank, as well as by direct user submissions. OpenFluDB has a rigorous curation process for all virus sequences, including those retrieved from GenBank.

FAO’s Emergency Prevention Programme for Transboundary Animal Diseases (EMPRES) has developed a Global Animal Disease Information System (EMPRES-i) to collect comprehensive epidemiological records for transboundary animal disease events, including influenza, and make this information available to the scientific community (16–19). EMPRES-i is a web-based application that enables to collect, store and analyze information from multiple sources, such as FAO projects (for disease surveillance in particular) and reports, the World Organization for Animal Health (OIE), official government sources, the European Commission, FAO reference centers and other laboratories and technical partners. Zoonotic influenza human cases are entered into EMPRES-i based on official sources (national authorities and the World Health Organization). Information is searched, scanned and entered on a daily basis and epidemiologists routinely validate entries into the database. Extensive effort is made to verify unofficial information and seek confirmation by national authorities. Only officially confirmed nonconfidential information is displayed to the public.

The EMPRES-i public interface is composed of a disease event component, a CVO directory (contact details for Chief Veterinary Officers), a laboratory directory (listing FAO and OIE reference centers, veterinary public health experts and laboratory networks) and a library (publications and images). The genetic module described in this manuscript is part of the disease event component. EMPRES-i records disease event data and disease surveillance data and enables data analysis and mapping. Mapping and graphic functions are available in the disease event component, with easy access to and retrieval of information on animal disease events worldwide, according to criteria selected by the user. Layers from other FAO databases can be added to the maps, such as data on livestock populations, human demographics, biophysical conditions and animal health status. The graph component enables users to produce simple line, bar and pie charts according to such variables as disease, serotype, time and number of outbreaks. To support this analysis, EMPRES-i integrates data and GIS resources from other FAO databases and systems, i.e. livestock density or environmental layers (e.g. the Global Livestock Production and Health Atlas, GLiPHA (20) available from Geonetwork (21) and from external systems [e.g. Bioportal (22)].

Since 2004, EMPRES-i organizes all available epidemiological data on specific animal disease events as comprehensive records and assigns individual event identifiers (id). An influenza event in the EMPRES-i public Web site is characterized as a nationally confirmed influenza finding in animals or humans (note: currently human cases are registered for H5N1 and H7N9 only). The information originates either from public sources (e.g. on the OIE Web site or in scientific journals) or from reports to FAO through official channels. Influenza events include outbreaks in animals on farm, village or commune level, cases in wildlife or humans or positive surveillance findings in animals. The availability of epidemiological information can greatly vary for such events as does the level of detail. Ideally, EMPRES-i disease event ids would be included in a genetic sequence record submitted to repositories like GenBank so that related epidemiological and genetic information could be identified and linked automatically. However, this systematic linkage will hardly be implemented because epidemiological and genetic information of disease events in animals follow different processing and validation pathways; the EMPRES-i disease event id is currently assigned after these paths have long parted. Moreover, huge numbers of influenza sequence records and large numbers of influenza outbreak records make it extremely difficult to restore these relations (links) between isolates and disease events without computational methods. In addition, tools to evaluate these links are currently not readily available. Often, the epidemiological information submitted along with genetic sequences to genetic databases such as GenBank or OpenFluDB is scarce and limited due to nonstringent rules applied in the submitting process and unawareness of submitters regarding the added value of such information; the minimum associated epidemiological information required while submitting a genetic sequence is year of sampling, country and species.

The aim of this article is to describe the tool for integration of influenza epidemiological data and sequence information, and to suggest possible applications of the genetic module for the influenza scientific community.

## Description and implementation of the matching algorithm

**Figure 1.**
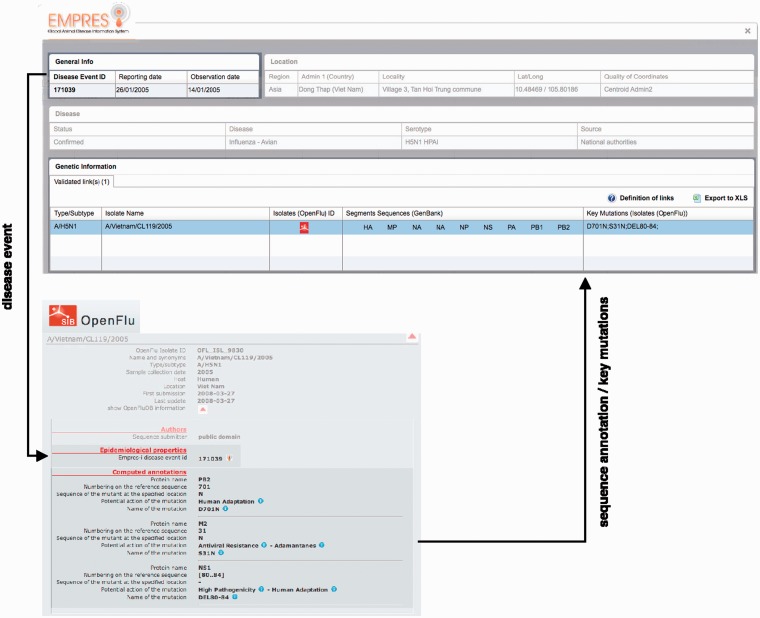
Linking EMPRES-i and OpenFluDB has implied the development of web services (terminology—query) for data comparison, the creation of an algorithm as the interface between both databases and enable the exchange of data (no transfer).

The linkage between an influenza event in EMPRES-i (id) and an influenza virus isolate in OpenFluDB is made by comparing three parameters: date, geographical location and host species, guaranteed to exist in both disease event and isolate record (Figure 1). Links can be queried and proposed in two directions: (i) for any event in EMPRES-i, a list of sequences with matching parameters will be proposed from OpenFluDB; (ii) for any sequence in OpenFluDB, a list of events with matching parameters will be proposed from EMPRES-i. The two databases are programmed to exchange necessary information and run matching on a daily basis, with subsequent manual validation of the suggested matches by FAO experts.

### Compatibility between EMPRES-i and OpenFlu databases

Matching influenza events referenced in EMPRES-i with virus isolates referenced in OpenFluDB requires concordance of annotations at the level of time of outbreak reporting and sample collection, at the level of geographical location and at the level of host taxonomy. The latter two levels are described differently in the two databases. A preliminary work has thus been performed to create associations between the two sources.

To geographically locate an event, EMPRES-i refers to GAUL identifiers (23) while OpenFluDB uses Geonames identifiers (24) for country, administrative division (state) and administrative subdivision (county) identification. Therefore, a procedure to match GAUL and Geonames identifiers based on name, synonyms and geographical coordinates was established and integrated into OpenFluDB. All countries were mapped between the two geographical systems used in EMPRES-i and OpenFluDB, but only 86% of administrative divisions (provinces) and 28% of administrative subdivisions (districts) in EMPRES-i could be mapped to the corresponding administrative divisions and subdivisions in OpenFluDB, respectively. However, whereas most of the EMPRES-i disease events have geographical information at administrative division level (100%) and administrative subdivision level (94%), only 63% of OpenFluDB isolates can be located at the administrative division level and only 5% at administrative subdivision level. Thus, the poor matching percentage at the subdivision level, does not impact too many computed links.

OpenFluDB and EMPRES-i also have different taxonomy classifications. The two classifications were compared, equivalent levels were identified and host species names used in EMPRES-i records were entered when necessary in OpenFluDB as synonyms for the existing taxons. This preparative work has allowed direct data comparison of the three parameters (date, geographical location, host species) between the two databases.

### Data exchange between the two databases: generation and scoring of disease event-isolate links

**Figure 2.**
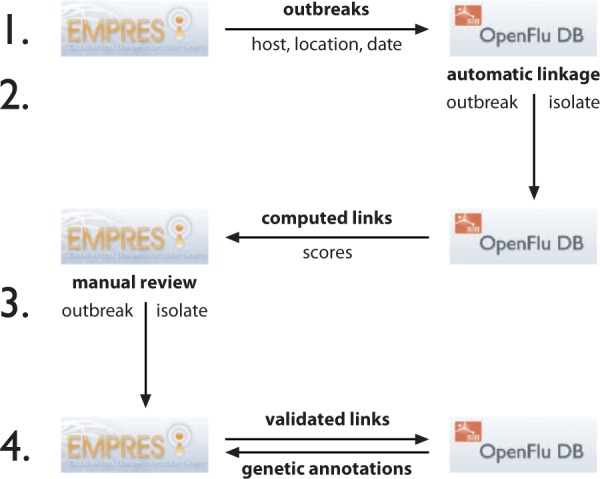
Flowchart showing steps to synchronize the linkage between EMPRES-i disease events to OpenFluDB isolates. The procedure has four steps and is based on web services (WS) to exchange information between both databases. (1) OpenFluDB gets a list of disease events described by host, location and date from EMPRES-i. 2) A procedure runs on OpenFluDB to assign scores to computed links between EMPRES-i disease events and OpenFluDB isolates. The computed links with scores are then communicated to EMPRES-i. 3) FAO experts validate the computed links. 4) The validated links are back-communicated to OpenFluDB to be shown as URLs to EMPRES-i on the corresponding OpenFluDB isolate record pages. EMPRES-i queries OpenFluDB to get genetic information about linked isolate(s). All these steps are run daily.

The linkage procedure is performed in four steps (Figure 2).

1) OpenFluDB queries EMPRES-i through a web service to synchronize the list of confirmed disease events as records of event id, observation date, host name and geographical location (GAUL id).

2) Computed links between each EMPRES-i disease event and their best-matched OpenFluDB isolates are produced with an automatic procedure. Each computed link is associated with a confidence score. This score is the weighted sum of three scores reflecting the similarities of geographical locations, host species and timing of sample collection. Geolocation tree matching is done on four levels: country, administrative division, administrative subdivision and geographical place (village or city). A maximum location score is given for an unambiguous association at the lowest geographical location level. The more ambiguous the association (e.g. when an association is possible only at country level or when a location is misspelled), the lower the score. Similarly, a successful match of hosts at species level yields a higher score than a match only at class level. Because a certain delay may exist between the sample collection date and the event observation date, the time score is decreased according to the difference between the event observation date and the sample collection date. The time score is additionally decreased to penalize situations when an isolate has a collection date before the observation date of a putatively associated event. However, we found that some tolerance regarding the time frame is still useful to deal with possible inaccuracy in reporting of either event or sample collection date. The same approach is necessary when the exact day of sample collection is not known, or the (possibly delayed) event reporting date is used instead of the exact observation date, considering that precise information for the latter is not always available. The minimal set of criteria to consider a putative match is same country, same animal class and same year. This sparse set of criteria has the consequence that for most of the outbreak events, the algorithm will suggest to link several isolates. However, because the sample collection dates and/or the host species and/or the sample collection location of those isolates might be more or less precise, the computed links of the suggested isolates will have different scores. We suggest that the most probable valid link will be the one with the higher score (or having rank one among the sorted list of computed scores). We represent this assumption by computing the confidence score of a match, which is obtained by dividing the total score value by the rank value.

3) EMPRES-i synchronizes with OpenFluDB the list of event-isolate computed links yielded from the last run of the matching procedure (new links are registered, while retired links are deleted). The links are stored in EMPRES-i, and subsequently reviewed by FAO experts using the confidence scores as initial guidance. Experts will evaluate validity of the computed links based on the additional epidemiological knowledge that is available in the EMPRES-i database, or through contacting FAO field offices, academic partners and sequence submitters to obtain additional info to make a valid linkage.

Three levels of links are distinguished in the EMPRES-i genetic module after expert judgment of the computed links by the algorithm or links suggested by scientists: (i) ‘Validated link’, the link between disease event and isolate(s) has been manually validated by FAO based on three linkage criteria: location matching, timing matching and species (host) matching, as well as on eventually available additional evidences; (ii) ‘Proposed link’, a link between disease event and isolate(s) that is considered but cannot be fully validated because of incomplete evidence. Proposed links can evolve to validated links, as new information is made available and/or suggestions are made by scientists directly involved in the analyses on the outbreak. (iii) Not available link, this category includes all those links that have been rejected by the experts, cannot be validated or proposed or merely do not exist, i.e. where no virus has been sequenced during an outbreak or when the sequence has not been made publicly available by the laboratory or has not passed the quality check in the OpenFlu curation system.

4) The validated and proposed links are shown through the EMPRES-i genetic module. The validated links are also communicated back to OpenFluDB, where an URL to the corresponding epidemiological information in EMPRES-i is presented on the isolate page.

The procedure of matching calculates all computed links from scratch on each run, thus accounting for eventual updates of both disease events and isolate records. The new set of links is then compared with the previous one. All new computed links are integrated, and the old links absent in the new set are marked as retired, both along with timestamp. These updates are communicated to EMPRES-i on a synchronization request. The statistics on automatic linkage is as follows: 90% of OpenFluDB H5N1 isolates (collected in 2004 or later) and 58% of H5N1 disease events available in EMPRES-i have at least one computed link suggested by the algorithm.

### Evaluation of the algorithm and refinement of the scoring system: linking H5 influenza events and genetic data

During a pilot study, an FAO expert evaluated the computed links proposed by the automatic matching procedure. The expert took the decision on validation of these links through comparison of the same three criteria: event location, species and date with the respective information of the sequences present in OpenFluDB. In difficult cases, additional expert knowledge, e.g. from national and international reference laboratories (in particular those that had deposited the sequence) was sought to approve (or reject) the proposed links.

**Figure 3.**
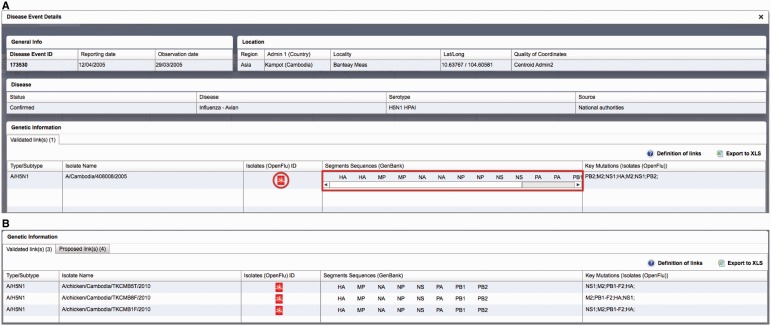
Example of epidemiological Record of Disease Event in EMPRES-i including validated links to genetic data: (**A**) with one linked isolate, and (**B**) with several linked isolates (validated and proposed), several sequence records and identified key mutations. Selecting the SIB icon 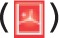 or individual links under ‘Segments Sequences’ for GenBank will open a new browser window or link to display the sequence record.

Critical assessment of the computed links proposed by the algorithm for HPAI H5N1 isolates reported between 2004 and 2012 resulted in the validation of 774 links out of 4446 H5N1 isolates present in OpenFluDB (17.4%). Among these links, 584 acquired the status of ‘validated links’ and 190 the status of ‘proposed links’. These 774 links correspond to 774 isolates and 537 EMPRES-i disease events. The higher number of linked isolates compared with linked events results from the fact that more than one virus isolates may be obtained from one outbreak in animals (Figure 3).

**Figure 4.**
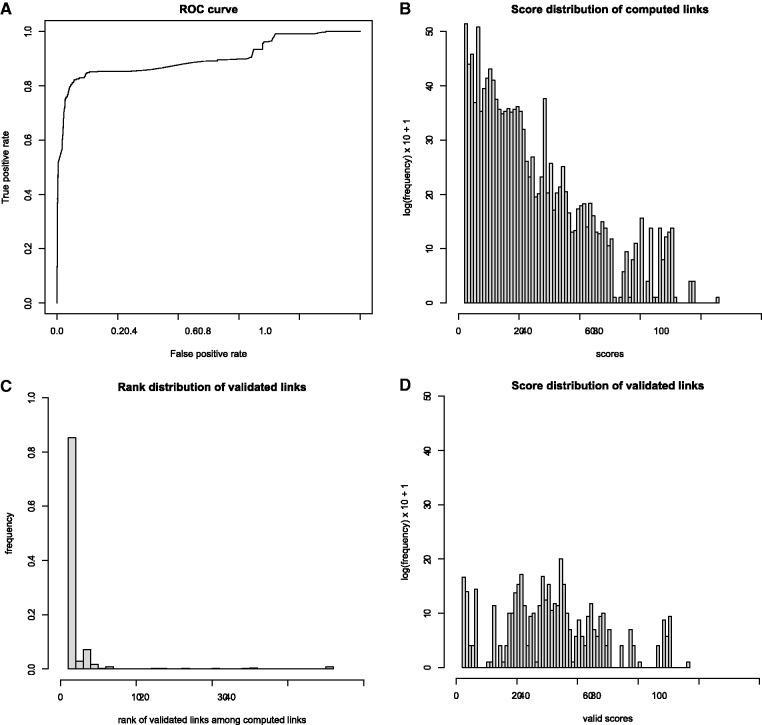
Evaluation of automatic matching by statistical comparison of all computed versus validated or proposed link scores. (**A**) ROC curve of true positive vs. false positive rate after optimizing the scoring system with a linear discriminant analysis. (**B**) Log frequency plot of 323 234 computed link scores. Each event-isolate link satisfies the three minimal linkage criteria: same country, same animal class, known year and month of the collect date, date delay ±18 days. Each score is a weighted sum of 3 subscores showing quality of match for species, geographical location and date, divided by the rank of this disease event among all other disease events linked with the same isolate. (**C**) Distribution of ranks of isolates in the expert approved (validated) links. Eighty-five percent of validated links are between an event and the top-ranked isolate. (**D**) Distribution of validated link scores. The validated links have significantly higher scores than all computed links (mean scores of 28.5, respectively 6.9. *P*-value of 2.2^e-16^ with a Wilcoxon rank test).

At the next step, this set of expert-validated links is used to evaluate the discrimination power of each individual component (geographical, host and timing) of the total score. This is done by applying a linear discriminant analysis to optimize the weights of those components to separate validated links from nonvalidated links. The result of this analysis shows that the matching at the level of the geographical place (village or city) has the most discriminant power (coefficient of discrimination of 0.39), followed by the matching at the geographical administration subunit level (coefficient 0.12), then matching at the geographical administration unit level (coefficient 0.05). The matching at timing level has a low discriminant power (coefficient 0.03). Finally, the matching at host level is poorly discriminant (coefficient 0.002). The discrimination power of this scoring system is illustrated with a receiver operating characteristic curve in Figure 4A. This plot illustrates that the scoring system has a good specificity but not a high sensitivity. Using those scores, experts can confidently select links that are likely to be validated.

The distribution profiles of all versus validated/proposed links were examined. Figure 4B shows the distribution of scores associated to all computed links. The set of computed links that are most likely to be validated are the one with an exact match at the host species level, a geographical location match at subcountry level (administrative unit) and a match at the month level of sample collection date/outbreak event reporting date. Considering the list of computed links that fulfill those stringent criteria, the lowest computed score between an outbreak event and its best linked isolate is 31. However, most of the scores are between 0 and 31. The computed links with the lowest scores (between 0 and 7) have exact matching at the species level but poor matching at the geographical location and timing levels; these associations are unlikely to be validated by an expert. The links that contain matching at the species level and timing level, but poor information at the geographical location level have scores ranging from 7 to 22. This is often due to missing details of the geographical annotation in OpenFluDB. The links that contain good matching at the location level and timing level but poor matching at the species level have scores between 16 and 34. Some valid links might be part of these last two populations, but their validation would require some additional information that might be available either in commentary fields of GenBank records or directly from the isolate sequence submitter. It is suggested that the computed links with a score >31 would contain a higher proportion of isolates really collected during the corresponding outbreak in animals.

Figure 4B shows that 85% of validated links have been made between an isolate and outbreak disease event with the highest score (rank 1). In the case where multiple isolates are linked to the same outbreak (e.g. outbreak 129 547 in Table 1), some pairs are established with isolates at rank ≤2. By comparing Figure 4B and D, one can observe that validated scores (mean score of 28.5) are significantly higher (Wilcoxon rank sum test: *P* = 2.2^e-16^) than all computed scores (mean score of 6.9).

**Table 1.** Three examples of validated links between influenza events and virus isolates

Unique Identifier (oubreak at isolate level)	ID	Country	Adm. division	Adm. Subdivision	Geo. place	Date	Host	Rank	Total
Outbreak	125796	Russian Federation	Rostovskaya Oblast	Gulyay-Borisovka, Zernogradskiy		12 December 2007	Chicken		
Isolate	OFL_ISL_14419	Russia	Rostovskaya Oblast'		Rotov-on-Don region, Zelenogradsky district, Gulyai-Borisovka	14 December 2007	Chicken	1	
Score		100	100	0	55	93	100	1	78
Outbreak	165752	Cambodia	Battambang		Opong Moan	3 November 2011	Chicken		
Isolate	OFL_ISL_65739	Cambodia	Khett Batdambang			3 November 2011	Chicken	1	
Score		100	100	0	0	100	100	1	36
Outbreak	129547	Thailand	Uthai Thani	Ban Rai	Village 6	10 November 2008	Chicken	1	
Isolate	OFL_ISL_35854	Thailand	Changwat Uthai Thani			14 November 2008	*Gallus gallus*	1	
Isolate	OFL_ISL_35852	Thailand	Changwat Uthai Thani			15 November 2008	*Gallus gallus*	1	
Isolate	OFL_ISL_35853	Thailand	Changwat Uthai Thani			16 November 2008	*Gallus gallus*	1	
Isolate	OFL_ISL_35855	Thailand	Changwat Uthai Thani			17 November 2008	*Gallus gallus*	1	
Score		100	100	0	0	100	100	1	35

The table contains the following columns. ID: EMPRES-i disease event and OpenFluDB isolate unique identifiers. Country: name of the country where the event occurred and where the isolate was collected. Adm. division and Adm. subdivision: geographical administrative division and subdivision where the event occurred and where the isolate was collected. Geo. place: geographical place (city, village, etc) where the event occurred and the isolate was collected. Date: date of event observation (or reporting, if observation date is unknown) and isolate collection. Host: host species affected by the disease and from which the isolate was collected. Rank: the rank is the position of a given link in the sorted list of computed links between this isolate and all events that could be associated, considering the minimal linkage criteria of same month and year of reporting, same country and same animal class. The total score is a weighted sum of all individual scores divided by the rank. One event can be linked to several isolates, as shown in the third example, but one isolate cannot be linked to multiple events.

Table 1 presents the scoring system with three examples of manually validated links. In the first example, while matching EMPRES-i disease event ID 125796 to isolate OFL_ISL_14419, the geographical component of the total score have been computed based on partial text matching with the administrative subdivision of the EMPRES-i record. The isolate has been collected two days after the event observation date resulting in a score of 93 at the date level. The total score is a weighted sum of all score components with a maximum of 100. In the second example, annotations at the country, administrative division, date and host perfectly matched but no information was available at the administrative subdivision level in the OpenFluDB record, resulting in a total score of 36. In the third example, several isolates have been sampled during one outbreak. They are all linked with the same score of 35.

Finally this analysis shows that the scoring model of the algorithm provides good separation power and is able to discriminate the valid from unrelated event-isolate links. However, as there is a possibility of having wrong suggestions among the high-scored links (false positives), critical evaluation of the linkage by experts will always be required.

## Discussion

### Proof of concept

In this article we present a proof of concept for a genetic module developed at the interface between EMPRES-i and OpenFluDB, able to link influenza genetic and epidemiological data. Such an approach of knowledge federation, where data are organized into domains of expertise, maintained at their origin and served to the community is much practiced nowadays. This approach ensures access for users to the most correct and complete body of data. However, the data accuracy directly depends on accuracy of primary data submitted by contributors, although efforts must sometimes be carried out to improve their quality and recuperate missing information. As an illustration, for an isolate record containing only minimally required epidemiological data (species, country and year) (25, 26), it is impossible to determine the matching disease event with reasonable confidence. The minimum data for proposing the link by manual procedure has been found to be location at province level, time with year and month and host species name. These categories are sufficient in a context where the number of outbreaks is limited in space and time. In the case of many outbreaks in the same species and location or where information on admin 1, month and/or species is not supplied together with the virus sequence, further information or country support is always needed to clarify the origin of isolates. Any ambiguity in two out of the three matching criteria increases the number of putative matches in such extent that expert contribution is most often required. On the contrary, when the day of sampling, the county-equivalent administrative subdivision (or the unambiguous town name, or the GPS coordinates) and the exact species are known, the matching procedure results in a single or just a few high-score matches. This illustration highlights the importance of a complete and accurate record of basic epidemiological information submitted along with genetic sequences. Optimally, inclusion of the EMPRES-i disease event id into the genetic sequence record (e.g. as a structured comment in GenBank) would immediately provide unambiguous linkage and abolish necessity of linkage through the algorithm and subsequent manual validation.

### Improving the linkage

One of the first challenges faced when trying to link the two databases was that they were not using the same references for describing host taxonomy and geographical locations. It was therefore initially not possible to establish an exact correspondence between the related references. Whereas for taxonomy, the work at NCBI Taxonomy appears to be widely used to identify species, no standard geolocation system is available. OpenFlu uses Geonames, a free-access geographical database, while FAO uses and manages GAUL (which is also freely available and uses part of Geonames) in all FAO projects. Because the most relevant information to link an outbreak event and an isolate is the geographical location, the concordance of the reference databases is crucial.

Although our analysis shows that the scoring model possesses good separation power, it cannot avoid generating some high-scored false-positive links. This means, that at the current state, the computed event-isolate links cannot be blindly accepted by EMPRES-i, even with a high score threshold. That, in turn, means necessity of critical evaluation of the linkage by one or more experts. By the time of writing, the validation could only be done on a limited number of events for expert labor is a precious and limited resource. This suggests widening the expert panel to (i) multiply manpower and (ii) expand the panel’s country-specific expertise. This might be achieved by engaging national experts (notably OFFLU contributors) and by promoting communication between local veterinary services and sequencing laboratories. In addition to the manually validated and proposed links, it is also necessary to keep records of rejected links, especially those among the high-scored. Such a pool of rejected links would be a valuable resource to fine-tune the algorithm and rules.

Despite significant investigation efforts made by FAO to manually validate links, only 13% of HPAI H5N1 isolates recorded in OpenFluDB have been linked to an EMPRES-i disease event (as of September 2013). Many links have been missed because of a lack of information within the matching criteria, especially in endemic countries given the high number of possible matches. Many other links have been missed in situations where the isolate has not been generated from a reported outbreak but from active surveillance; in this situation, no EMPRES-i entry may exist. This gap is being addressed by entering published positive results of active surveillance into EMPRES-i. However, many potential links are lost forever, which represents a data loss. Efforts will continue to validate links between the highest possible proportion of available influenza virus sequences with outbreaks present in the EMPRES-i disease event component and with active surveillance events described in reports and scientific papers. It is also hoped that scientists and technicians will contribute to the efforts made by FAO, by suggesting or confirming links between virus sequences and outbreaks/discoveries during active surveillance. An interactive system has been put in place in EMPRES-i for these contributions. Sustained education and awareness raising on the importance of recording associated laboratory and epidemiological data is required, as well as better collaboration between laboratory and epidemiological teams. Cultural barriers can be overcome if approaches are developed to integrate virus related factors into risk analyses/modeling. Export functions of combined epidemiological and sequence information should support these efforts.

So far, linking efforts have focused on H5N1 and avian influenza A(H7N9) isolates (2013), which have been the most salient zoonotic influenza in the past years. This genetic module will have more value if other subtypes of zoonotic significance are also linked, yet these subtypes are so far not readily entered in EMPRES-i.

This initiative represents the first step of collaboration between two databases containing influenza data. It can potentially be expanded to a network of collaborations between EMPRES-i and others influenza-specific databases who would be willing to implement and run the algorithm described. This extension would presumably have a star-like architecture, where EMPRES-i polls its partners one-by-one, withdraws the newly evaluated links, stocks them and optionally manages identical links received from different partners.

As more validated, proposed and rejected links are recorded, the scoring procedure might be refined to better help the curators work. However, with current annotation quality and completeness of genetic records, a fully automated linkage mechanism will certainly associate incorrect isolates with an outbreak, thus potentially leading users of the EMPRES-i genetic module to draw erroneous conclusions on the reported observations. This problem might be solved if the submitters of the genetic sequences associate the corresponding EMPRES-i disease event identifier on their submission to GenBank or OpenFlu.

### Applications

Combining surveillance, genetic characterization and geomapping for animal influenza viruses will contribute to the work of epidemiologists, virologists and disease ecologists. It is to be noted that this genetic module benefits from tools and data already present within EMPRES-i (for spatial mapping, export of information and interfaces with other databases, such as GLiPHA) and within OpenFluDB (phylogenic tools). In particular, Sequence Similarity Maps (SSM) enable identification of relative genetic distances for a high number of viruses (14). These SSMs can be used to study virus evolution and clusters, and may be combined with epidemiological information (e.g. date, species, location) to create spatiotemporal maps of virus clades and clusters that can be overlaid by other layers such as animal densities maps. Screening for distribution of viruses (according to geographical location, host, time) with molecular markers of interest, identification of potential drivers of virus evolution and the occurrence of reassortment events and knowledge gap analysis of virus sequences are other possible applications. Adding information such as agroecological farming system characteristics to spatiotemporal information on influenza circulation can also support risk modeling of influenza emergence and spread in animal populations, and possible transmission to humans, by refining risk factors and specifying influenza high-risk areas.

This module can therefore offer advanced analytical tools for processing information and providing geographical mapping and spatiotemporal distribution of gene pools, clades or clusters, or molecular markers, and also overlaying genetic information and epidemiological risk factors such as animal population densities or production systems, tracing disease events with similar genetic characteristics, screening for disease events that involve viruses with certain molecular markers and more in the future, according to identified needs.

This module is currently available to the public. It is expected that scientists, both epidemiologists and virologists, and even decision makers, will make use of it to map combined information and to perform (risk) analysis, especially using the mapping and export functions (27, 28). It has already been used by FAO to support risk assessment of human–animal influenza threats for the H7N3 influenza outbreak in Mexico (29), and has been endorsed by OFFLU, the OIE/FAO network of expertise on animal influenza (30). In the future, this tool can potentially be used for OFFLU’s technical contribution to the WHO Vaccine Strain Selection Process, biannual expert consultation to evaluate circulating influenza strains and select candidate viruses to serve as seed strains for the development of pandemic vaccines. It may also support the influenza gene observatory, a proposed approach to monitor possible influenza gene and virus progenitors of future strains with pandemic potential. This module can capitalize on existing achievements and investments to develop a global influenza surveillance network and a permanent observatory, including spatial and temporal data, which will improve the understanding of the evolution dynamics of the influenza virus gene pool in animals and humans.

## Conclusions

Integration of viral characteristics into an animal disease database such as the EMPRES-i genetic module provides a unique tool to improve disease epidemiology and ecology knowledge. This interface will allow for deeper analysis, including risk modeling, transmission pathways and ecological analyses, based on tools already developed and integrated in EMPRES-i and OpenFluDB. It should be emphasized that the data present in EMPRES-i and influenza sequence databases imply a tremendous field work and the willingness of countries to collaborate and share their disease and pathogen information with the international community. Education on the importance of data quality (precision and accuracy) remains crucial for a successful linking process in the future. Development of this module for other diseases is being considered, and has already been initiated for foot-and-mouth disease.

## Implementation details

### Web services

Two SOAP-based web services were developed on both sides to exchange data between EMPRES-i and OpenFluDB. The first web service developed by FAO enables OpenFluDB to query the EMPRES-i database and retrieve a set of disease events for a given virus subtype and date. This web service accepts queries by either a period when the event happened, or by a date of last synchronization between the EMPRES-i and OpenFluDB databases. The second web service, part of OpenFluDB, aims at serving OpenFlu isolates putatively linked to EMPRES-i disease events. Its main method takes as input either a period of the event date or the last synchronization date, as well as the serotype involved (all parameters are optional). This procedure returns an array of EMPRES-i disease events, each linked to one or several OpenFlu isolates. A companion method shows number of links to be synchronized. The third method integrated in OpenFluDB takes as input an EMPRES-i disease event id and returns an array of matching OpenFlu isolates together with their records of properties, like computed mutations. Yet another method at EMPRES-i concludes the data exchange according to the Figure 1 by providing all validated links as of a requested synchronization date.

These web services are not publicly available.

### Matching

Matching is performed by ranking of all suitable isolates against all EMPRES-i disease events according to the best combination of the three criteria: geolocation tree match, species tree match and time difference. Similarity on each of the three criteria is evaluated as numeric scores, with the total score as a sum.

Geolocation tree matching is done on four levels—country, admin1, admin2 and geoplace using either direct match or flexible (i.e. spelling-error tolerating) word search algorithm with max score of 100 for the exact match. Partial scores are evaluated for each level, except for the country level where exact match is required. To match species, the OpenFlu's species tree of unregulated depth (current max depth is 6) is dynamically reduced to FAO's two-level species tree, and some additional synonyms were introduced. Match on species level is awarded by 150, on the class level by 50. Date mismatch score is modeled by an exponential decay function using the days mismatch value as its argument. In the percent scale, this score component ranges from 100 (no mismatch) to ∼0 after 18 days of difference. If exact day is not known, its value is arbitrary assumed to be 14th, then proceeded as usual, but the max score is set to 30 instead of 100.

After the main run of matching resulting in a set of scored isolate-outbreak pairs, isolate ranks (count of other outbreak events matching the same isolate with the same or a higher score) are calculated. These ranks are then used to correct scores for excessive cardinality by simply dividing the score value by the rank value. The total score is a weighted sum of all those partial scores. The weights are computed by performing a linear discriminant analysis on the set of validated/nonvalidated links (lda command of the MASS package of the R statistical software). Finally, normalization relative to the maximal possible value renders the score to its final user-friendly percent form (named in the manuscript as confidence score).

The web service on EMPRES-i is implemented in Java J2EE, SQL and SOAP. The matching and web service on OpenFluDB side is implemented in SQL and PHP.

## Funding

This work has been sponsored by the FAO project IDENTIFY (OSRO/INT/902/USA), funded by USAID under the Emerging Pandemic Threat Program as well as SIB by the Swiss Federal Government through the State Secretariat for Education, Research and Innovation. Computations were performed at the Vital-IT Center of the SIB Swiss Institute of Bioinformatics.

*Conflict of interest*. None declared.
